# Maximizing the information obtained from chamber-based greenhouse gas exchange measurements in remote areas

**DOI:** 10.1016/j.mex.2018.07.021

**Published:** 2018-08-22

**Authors:** Haifa Debouk, Núria Altimir, Maria-Teresa Sebastià

**Affiliations:** a Laboratory of Functional Ecology and Global Change, Forest Sciences Centre of Catalonia, Carretera de St. Llorenç de Morunys km 2, 25280 Solsona, Spain; b GAMES Group & HBJ Dept., ETSEA, University of Lleida, Avinguda Alcalde Rovira Roure 191, 25198 Lleida, Spain; c INAR, University of Helsinki, 00014, Finland

**Keywords:** Soil-atmosphere multi-gas exchange in remote areas, CO_2_, CH_4_, N_2_O, Detection limit, Quantitative and qualitative data

## Abstract

Measurements of greenhouse gas (GHG) fluxes, particularly methane (CH_4_) and nitrous oxide (N_2_O) in mountain ecosystems are scarce due to the complexity and unpredictable behavior of these gases, in addition to the remoteness of these ecosystems. In this context, we measured CO_2_, CH_4_, and N_2_O fluxes in four semi-natural pastures in the Pyrenees to investigate their magnitude and range of variability. Our interest was to study GHG phenomena at the patch-level, therefore we chose to measure the gas-exchange using a combination of a gas analyzer and manual chambers. The analyzer used is a photoacoustic field gas-monitor that allows multi-gas instantaneous measurements. After implementing quality control and corrections, data was of variable quality. We tackled this by categorizing data as to providing quantitative or only qualitative information:

• 50% and 59% of all CH_4_ and N_2_O data, respectively, provided quantitative information above the detection limit.
• We chose not to discard data providing only qualitative information, because they identify highest- and lowest-flux peak periods and indicate the variability of the fluxes, along different altitudes and under different climatic conditions.
• We chose not to give fluxes below detection limit a quantitative value but to acknowledge them as values identifying periods with low fluxes.

## Graphical abstract

**Figure:**
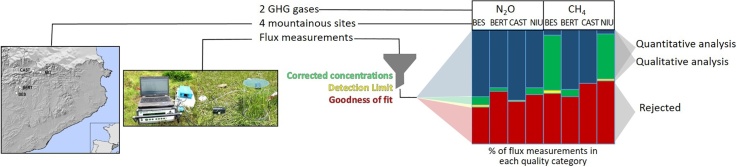

Specifications Table

**Table:** 

Subject area	• *Agricultural and Biological Sciences*
More specific subject area	• *Biogeochemistry*
Method name	*Soil-atmosphere multi-gas exchange in remote areas*

## Method details

To investigate the patterns of greenhouse gases (GHG) in extensively managed semi-natural grasslands in the Eastern Pyrenees, we measured vegetation and soil fluxes of CO_2_, CH_4_, and N_2_O from four grassland locations along an altitudinal gradient in the Eastern Pyrenees (Fig. S1 in Supplementary material). The locations are in remote mountain areas, with neither practical possibility of connection to the electrical network, nor capability for storage. The fluxes were measured intermittently during 2012 and 2013 with a portable gas-exchange system. Two of the four locations (BERT1276 and CAST1850) were equipped with eddy-covariance towers which provide continuous recordings of the photosynthetically active radiation (PAR), air temperature (T_a_), and soil water content (SWC) and thus describe the seasonal patterns and give a temporal context for the GHG flux campaigns, which covered the growing season. The altitudinal gradient is clearly reflected in the air temperature, with sites going from warmer to cooler with altitude. In the sites with SWC data, the low-altitude site presents the driest soil. Note that in 2012 there was a rather intense drought period that affected also the highest-altitude sites.

### System setup

**Fig. 1:**
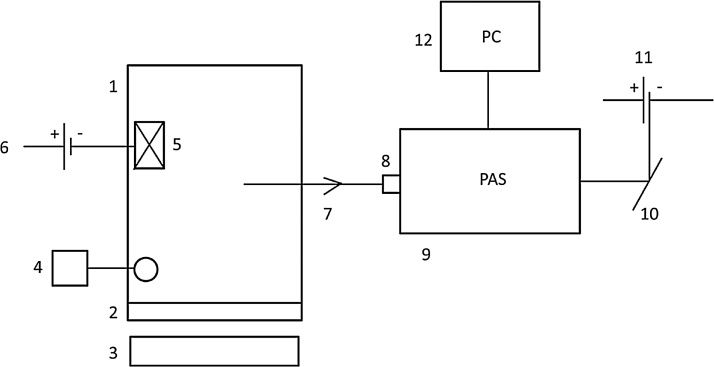
Scheme of the chamber-scale gas-exchange measurement system set-up. The enclosure consisted of (1) a methacrylate chamber (h = 38.5 cm; Ø = 25 cm), (2) a rubber joint at its base to provide sealing at the chamber/collar junction, and (3) a metal collar (h = 8 cm; Ø = 25 cm) installed 3 cm deep in the soil. The chamber was equipped with (4) monitoring of the internal air temperature with thermocouple connected to an AMPROBE multi-logger thermometer TMD-56, (5 and 6) air mixing to homogenize the air in the headspace with a small fan powered by its own battery, and (7) a 20-m long Teflon sampling tube. Air was intermittently drawn at a rate of 1 L per minute (LPM) and passed through an external air-filter before entering the (8) PAS analyzer (9) and being eventually exhausted. The flow rate was produced and determined by the analyzer, therefore there was no additional pump or flow controller. The system was powered by 12 V batteries +12 V–220 V converter (10 and 11). Communication to PAS and data storing was done with a laptop (12). To allow portability, the equipment was housed on a carriage and it was shaded from direct sun-light to avoid overheating.

We used a self–assembled portable gas-exchange system to perform *in-situ* field surveys. The use of PAS seemed to be a good alternative to do multi-gas concentration measurements due to its relatively high portability, ease of use, and low energy consumption [1]. The potential of this technology has been contemplated in a number of reviews of GHG chamber-based measurements [2,3]. The system consisted of a cylindrical chamber (20 l nominal volume), connected to a multi-gas analyzer through Teflon tubing. The chamber was made of uncoated transparent methacrylate that was darkened when needed with a reflective cover manually placed on its top (see details of the set up in Fig. 1).

We measured all gases simultaneously with a photoacoustic spectroscopy (PAS) analyzer (INNOVA 1412, LumaSense Technologies, Denmark). PAS has a measurement cycle that implies intermittent air flow from the chamber. The cycle starts by drawing air from the sampling point in order to flush the old air in the system and thereafter obtain a sample of fresh air. When the required volume of the sample is reached, the flow stops. From this, the concentration of the desired gases is consecutively determined inside the cell of the analyzer. The sample is irradiated in a modulated way to produce intermittent expansions, which can be detected photoacoustically. Each gas of interest is determined separately, as the irradiation is delivered through optical filters with selected wavelengths, and the filters are applied in sequence. The response time depends on the sampling integration and the flushing time defined; which in this study was approximately 60 s including the three gases and water vapor. This implies that the concentration output rate was of approximately one value per minute. The air removed from the headspace including flushing and sampling represents about 1% of the total chamber headspace. The removal happens during less than 10 s, leaving the system more than 50 s to replace the air -which will be homogenized by the small ventilator- before the next sampling volume is removed.

The nominal detection limits of the various gases are: 5, 0.03, and 0.24 ppm for CO_2_, N_2_O, and CH_4_, respectively. Prior to the field campaigns, the PAS was fully calibrated by the vendor [4] and taken into use in a plug-and-play basis with no need for recalibration during use, according to the recommendations of the vendor and as applied in other studies [e.g. 5,6]. Conforming to the instrument instructions, we used the analyzer in the cross-interference and the water-interference modes, to take into account the cross interference between gases and the interference of water vapor in the measure of gases (for more details on PAS modus-operandi and comparability see Iqbal et al. [6]).

The chambers were secured to the ground by fixing them to collars that are partially inserted in the soil. The collars were placed into the ground (3 cm deep) two to three weeks before each measurement period, in order to limit any disturbance in the soil prior to sampling. During measurements, the chambers were moved manually between sampling points. Flux measurements were done by placing the chamber around the collar to enclose the vegetation and soil for about four minutes. The chamber was always left open for four minutes before each flux measurement to ensure ventilation of the chamber headspace, and to obtain the values of ambient gas concentrations. We first measured fluxes of intact vegetation and soil under light, then under dark conditions. Afterwards we cut the aboveground vegetation and measured soil fluxes without vegetation and under dark conditions. Despite studies suggesting an increase in methane emissions [7] after plant removal, we did not observe any remarkably rising peaks of methane fluxes linked to this sampling effect.

### Flux calculations

**Fig. 2:**
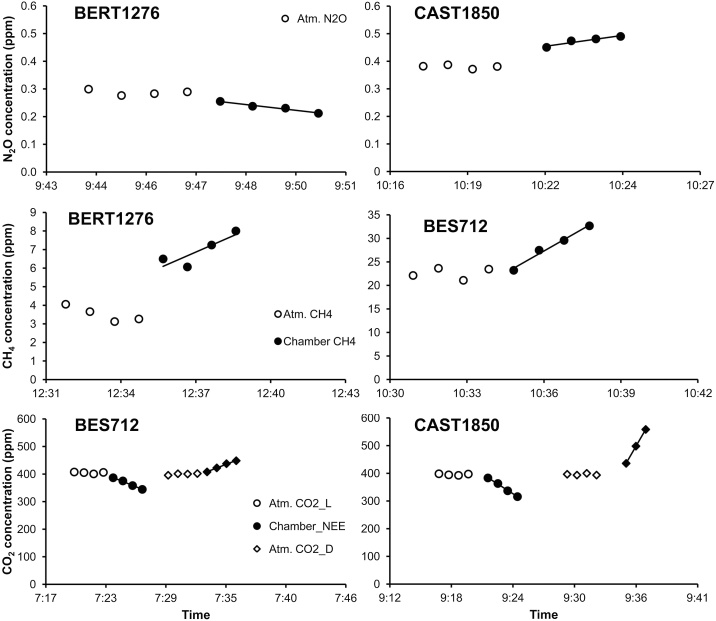
Examples of atmospheric/ambient and chamber closure concentrations of CO_2_, CH_4_, and N_2_O along time, showing single closures. Open symbols refer to the ambient concentrations measured before chamber closure, and full symbols are the concentrations inside the closed chamber. In case of CO_2_, examples are showed for both measurements under light (L) and dark (D) conditions; Chamber_NEE = net ecosystem exchange; Chamber_Reco = ecosystem respiration. The line corresponds to the fitted linear regression.

The flux was computed using the change in gas concentration monitored during the closure. When the chamber is placed in the collar, the plant-soil system is forced into a dynamic state where light, temperature, humidity and gas concentrations change due to the activity of the soil-plant system, which in turn responds to the change. The flux of interest is the rate of concentration change since the time of closure. This should preferably be estimated through a non-linear fitting procedure, as reported extensively [8] and references therein]. In our case, non-linear fitting was challenged by the small amount of measurements per closure, which makes several-parameter fitting spurious [9]. In addition, N_2_O and CH_4_ present small noise to signal ratio so that flux values rendered through calculation might not be statistically significant from 0 [10]. Therefore, we used a linear estimation (Fig. 2) to calculate the flux as the slope of the relationship of gas concentration versus time:

$$F=\frac{V\partial C}{A\partial t}$$

where F is the flux in mol/s, V is the chamber volume in m^3^, A is the chamber surface area in m^2^, *δ*C is the gas concentration in mol/m^3^, and t is the time in s. Positive flux values refer to gas emissions to the atmosphere, and negative values represent uptake of the gas by the vegetation and/or the soil. The obtained total measured flux is the net result of all the mechanisms generating a change in concentration in the headspace, that is, fluxes generated by the soil (*F_soil_*) and the plants (*F_plants_*) as well as potential artifacts of the measuring system (*F_system_*); which refer to the error margin that may occur in the system.

$$F_{measured}=F_{plants}+\;F_{soil}+\;F_{system}$$

### Quality control and corrections

**Fig. 3:**
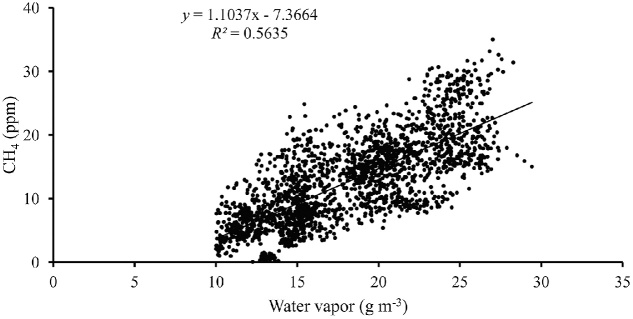
Relationship between CH_4_ concentrations (ppm) measured with the PAS from ambient air and water vapor (g m^−3^) for all the four grassland sites.

All collected raw data were screened for integrity and outliers. As the examination of the concentration data revealed a remaining interference of water vapor on CH_4_ concentration, a correction was applied similarly as in [11]. The slope between the concentrations of these two gases showed an increase of 1.104 per 1 mol/m^3^ of water vapor, and this happened at water vapor concentrations higher than 12 mol/m^3^ (Fig. 3). Correspondingly, the CH_4_ value was lowered by a factor of 1.104 proportional to the change in water vapor since the previous measurement.

The overall background noise of the system was assessed from the measurements of the ambient concentration as the standard deviation (SD) over the average. This was also used to estimate the flux detection limit (DL) over 4 min as:

$$DL\;=\frac{V\times SD}{A\times t}$$

where t is total closure time (in our case 240 s). The goodness of fit of the flux calculation was assessed from the r^2^ value.

**Fig. 4:**
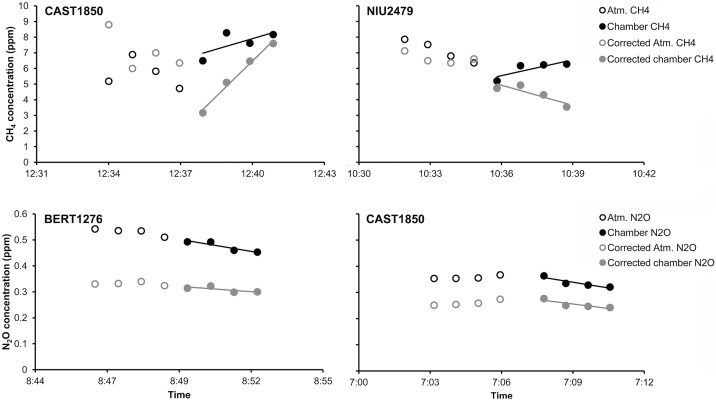
Examples of atmospheric/ambient and chamber closure concentrations of CO_2_, CH_4_, and N_2_O along time before and after corrections. Atm. CH_4_ = ambient CH_4_ concentrations; Chamber CH_4_ = CH_4_ concentrations inside the chamber; Atm. N_2_O = ambient N_2_O concentrations; Chamber N_2_O = N_2_O concentrations inside the chamber. The line corresponds to the fitted linear regression.

Temperature and relative humidity increase inside the chamber, especially during CO_2_ measurements on intact vegetation, were considered during chamber closure due to their effect on the stomatal behavior of the plants. In general, temperature increased in a range of 2–3 °C during chamber closure, reaching up to 5 °C in certain measurements days of the hot summer period, and the temperature change was considered during flux calculations. As for relative humidity, our inspection of the water vapor data before and after chamber closure (Fig. 2) showed no saturation of the system. Examples of modified concentrations of CH_4_ and N_2_O after the applied water vapor concentrations can be observed in Fig. 4.

### System performance

**Fig. 5:**
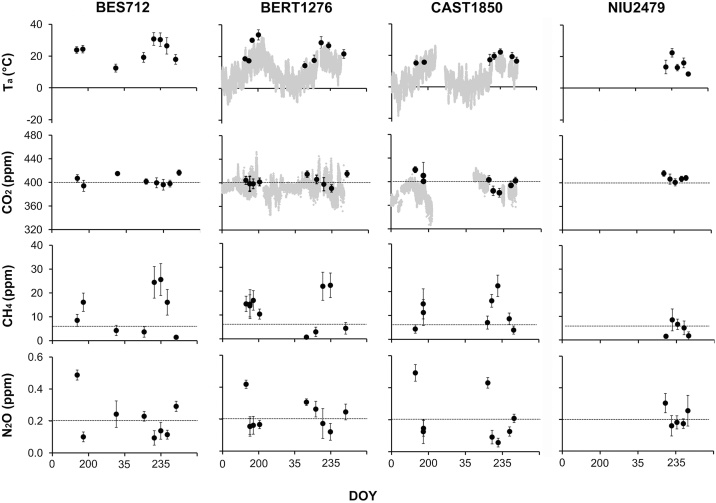
Temporal course (expressed in day of the year – DOY) of ambient measurements throughout the campaigns at the four grassland sites: BES712, BERT1276, CAST1850, and NIU2479. Values are daily averages and bars denote standard deviation (SD). In the case of BERT1276 and CAST1850, the continuous recordings from the meteorological station are also shown in the gray area: CO_2_ and air temperature (T_a_). The horizontal lines in the CO_2_, CH_4_, and N_2_O concentrations mark the global average ambient concentration for these gases.

A summary of the data can be seen in Fig. 5, which shows the temporal course of ambient measurements to give an overview of the environmental conditions, the sampling frequency and the comparability of the values between sites. It is also of interest to observe the level of ambient gas concentrations. In the case of CO_2_, we can see by comparison with the micrometeorological data that they were about the same level and followed the same temporal patterns (Fig. 5). The levels of N_2_O were in the order of magnitude of the background atmospheric concentration, 0.2 ppm, although spanning from 0.1 to 0.5 ppm. N_2_O seemed to be higher at the vegetation peak, and decreasing along the grazing season. An opposite pattern was seen for CH_4_, with the lowest concentrations shown around the vegetation peak. The lowest measured CH_4_ concentrations were in the range of the background atmospheric concentration, 2 ppm, whereas the highest values were an order of magnitude higher.

The overall background noise of the ambient gas concentrations measurements is reflected by the standard deviation (SD) of the mean, and can be seen in Fig. 5. We observed certain variability in SD between dates and between sites. Particularly, we detected temporal patterns of SD for all gases, with higher variability at the vegetation peak in comparison to very low variability during the early season or in autumn. This higher range of variability may be attributed to increasing temperatures and higher biological activities and emissions at the vegetation peak.

**Fig. 6:**
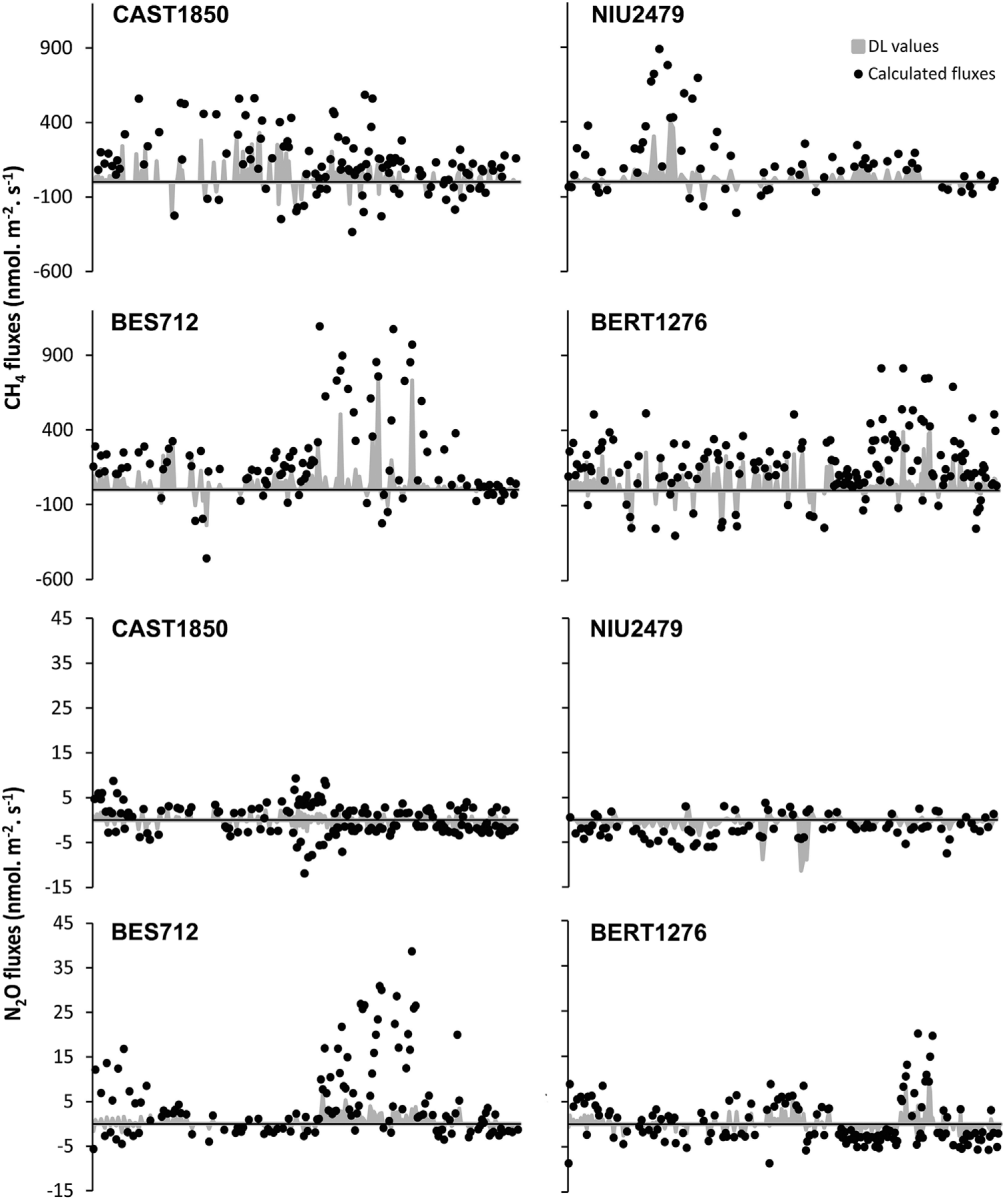
Comparison between the total calculated CH_4_ and N_2_O fluxes and the detection limit values (DL; gray-shaded area) in the four grassland sites along time, along the altitudinal gradient: Besora (BES712), La Bertolina (BERT1276), Castellar de n’Hug (CAST1850), Niu de l’Àliga (NIU2479).

The calculated DL of the fluxes changed according to the variability in the standard deviation. In the case of CH_4_, 53% of all measured fluxes exceeded DL in all sites, while 63% of all measured N_2_O fluxes exceeded DL (Fig. 6). In general, the lowest percentage of fluxes below DL was mainly observed during the autumn season, when the vegetation activity declines.

### Data treatment

To approach the variability in data quality we labeled the resulting flux values with quality flags as indicated in Table 1.

**Table 1:** Flagging codes with notes of usage, and criteria used for their description.

Flag usage		0	1	2	3	
		Absolute values useful for quantitative analysis	Some uncertainty for quantitative analysis	Only useful for qualitative analysis	Not useful	
Criteria	Correction needed	NO	YES	YES	YES	
	Good linear fit	YES	YES	YES	NO	NO
	Above DL	YES	YES	NO	YES	NO

Correction needed refers to whether the GHG concentrations required water vapor corrections post-measurements; good linear fit refers to whether the linear estimation of the GHG flux after a 4-min chamber closure has a good R-square value (>0.2) or not; above DL refers to whether GHG flux values are < or > detection limit values for each gas.

**Fig. 7:**
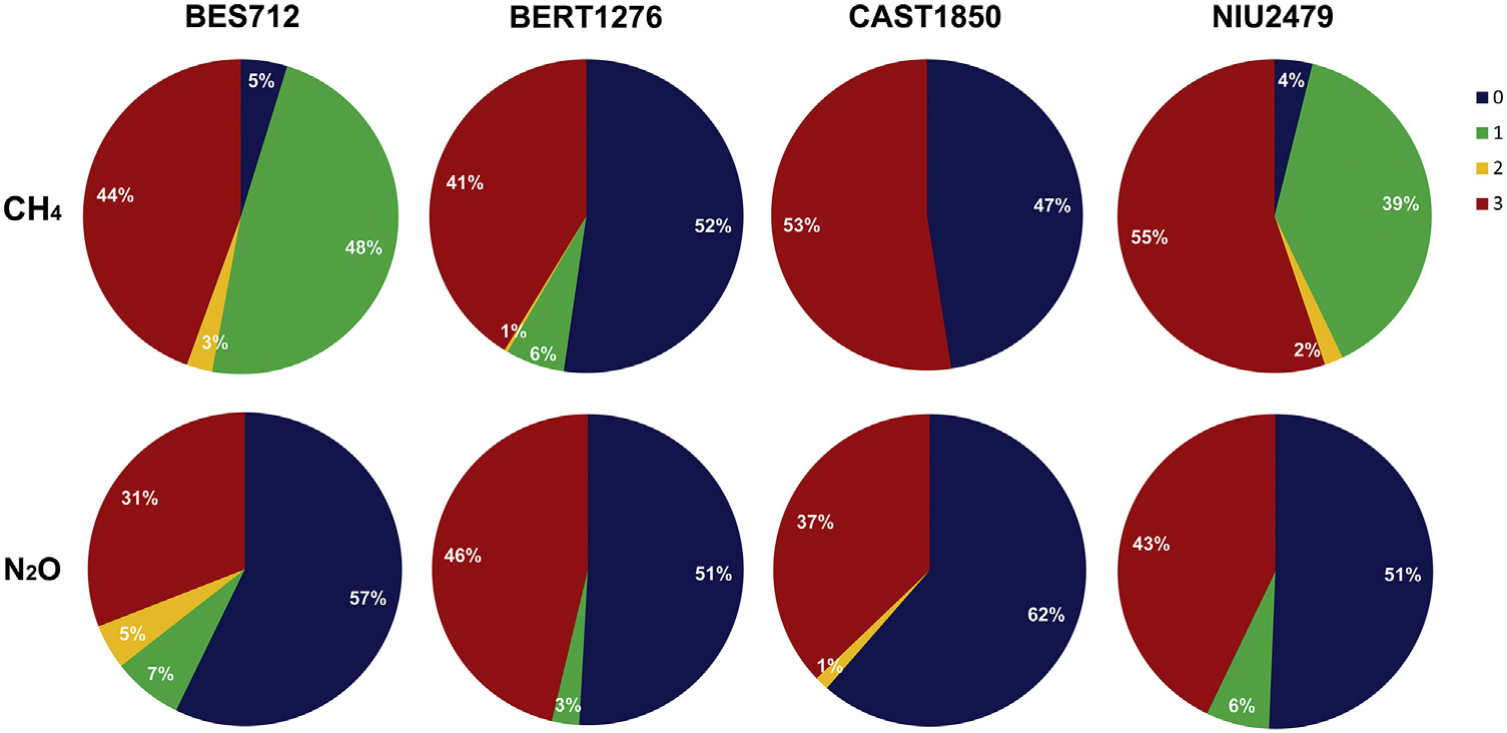
Pie charts presenting the percentage of the quality flags of all the measured CH_4_ and N_2_O fluxes in the four study sites: Besora (BES712), La Bertolina (BERT1276), Castellar de n’Hug (CAST1850), Niu de l’Àliga (NIU2479). For more details on the flags criteria (0–3), see Table 1.

In total, >50% of N_2_O data in all the study sites did not need corrections and thus belonged to flag 0 (Fig. 7). As for methane, we observed that the percentage of data needing further water vapor corrections (flags 1–3) was higher, particularly in BES712 and NIU2479 compared to BERT1276 and CAST1850 (Fig. 7). This is most likely due to the fact that more measurement campaigns were conducted during the years 2012 and 2013 in BERT1276 and CAST1850 in comparison to BES712 and NIU2479 (for more details on the experimental design and the gas measurement campaigns see supplementary material). However for N_2_O, data needing further corrections (flags 1 and 2) in comparison to those belonging to flag 0 were relatively consistent among all the study sites (Fig. 7). In addition to the flag differences according to the study sites, we also observed a temporal distribution of the flags where data corresponding to flags 1 and 2 occurred more in the year 2012 compared to 2013, and data belonging to flag 3 (very low fluxes mostly < DL) were more dominant in early or late vegetation period.

## Results and discussion

### Flux measurements with manual chamber and PAS

Several methods have been conducted in grasslands to measure GHG emissions; some use the eddy-covariance technique [e.g. 12,13] and others use chambers [e.g. 14,15]. We chose to use a combination of manual chambers and PAS analyzer, due to the great advantage of portability and multi-gas instantaneous measurements. A number of studies have already discussed methods and protocols for using manual chambers and PAS. Some studies considered PAS to perform reasonably well when compared with other methods for GHG concentration measurements [e.g. 6,16]. However, the system is limited to detect N_2_O and CH_4_ when their fluxes become too small at the sampling site, which was the case for 37% and 47% of our measurements of N_2_O and CH_4_, respectively. The rest of the fluxes were detectable and could be analyzed further (Fig. 7). Several studies aiming to measure soil fluxes of methane and nitrous oxide tend to use non-transparent chambers and close them for as long as 40 min [e.g. 17,18]. In our case, the vegetation presence in the chamber headspace made the closure time necessarily short (four minutes), similar to other studies using transparent chambers that chose a closure time of five [19] and three minutes [20] to avoid condensation. This short accumulation time was obviously limiting the capacity to detect the smaller fluxes, yet it allowed us to investigate and compare fluxes with and without vegetation, under light and dark conditions. Another possible reason for the low fluxes below detection limit may be the relatively large volume of the chamber used to cover the vegetation, which may have reduced the flux detection sensitivity [21,22].

### Variability in data quality

It is clear that for the data belonging to flag 0, no further correction was needed and fluxes were above DL and were calculated with a good linear fit, however sticking to these data and discarding data of flags 1 and 2 would make us lose important information on the GHG fluxes. For instance, data in the flag 2 allow us to identify the periods with the highest flux peaks and those with the lowest fluxes. Also, data corresponding to flags 1 and 2 provide us with a larger pool of data and thus more information about the range and variability of the fluxes in these grassland ecosystems, along different altitudes and under different climatic conditions. However, we believe that data belonging to flag 3 shall be discarded because fluxes with a very low R-square (those with R^2^ < 0.2) reflect a poor or non-linear fit and thus fluxes are not reliable for further statistical analyses.

The data quality levels do not vary randomly, but they seem to cluster around certain periods/seasons and circumstances. For instance, more N_2_O and CH_4_ data corresponding to flags 1 and 2 occurred in the year 2012 in comparison to 2013, and more data belonging to flag 3 (very low fluxes < DL) were dominant in early or late growth period. This can be attributed to the climatic conditions in these grasslands during these two periods, where more instability and fluctuations in terms of temperature and humidity usually occur. Also, the fact that more CH_4_ data needing further corrections (flags 1 and 2; Fig. 7) were observed in BES712 and NIU2479 is most likely because: 1) less (BES712) or no gas measurements (NIU2479) were carried out in 2012 compared to the sites BERT1276 and CAST1850, and thus the sample size and the data pool are smaller; 2) NIU2479 is an alpine grassland where humidity and wind fluctuations are most likely to occur, thus affecting the water vapor and its influence on methane measurements with the PAS analyzer.

Reporting flux values below detection limit or discarding them remains controversial. While some studies consider the flux values below detection limit unreliable and jeopardized, particularly for statistical analyses because of the lack of a numerical result [23,24], others regard these data as valuable, and deleting them or substituting their values with a zero or a constant value may lead to undesirable errors and misinterpretations [25,26], especially because they provide an insight on individual measurements and contribute to a better interpretation of the set of environmental observations. We chose not to give a quantitative value to measurements under DL, but to acknowledge them as very small values that identify periods with low fluxes.

The PAS analyzer has recently received attention [e.g. 11,16] concerning a potential cross-interference between gases, which is not sufficiently taken into account by the default settings, especially in unstable field conditions. Indeed, the PAS shows stable readings in the laboratory and in other stable environments such as farms and barns [[27], [28], [29]] but the environmental variability under field conditions in terms of temperature and humidity seems to compromise the precision of the measurements, in such a way that the baseline of the readings seems to drift in the field. For this reason, Rosenstock et al. [30] called for caution and reservation in its use. There are however studies that were able to take this into account [e.g. [31], [32], [33]] or used corrections *a posteriori* [11]. In our study, the interference of water vapor with CH_4_ was particularly obvious (Fig. 7). Therefore, we minimized a posteriori its effect on the flux calculation, by removing the virtual flux of CH_4_ created by the rising water vapor concentration during closure. Yet, it was impossible for us to fully get rid of this deviation in absolute numbers. However as the value of interest, in our case, is not the concentration of the gases per se, but the difference of concentrations in time -the flux- it is sufficient to consider the potential correction needed during a single flux measurement period, without being indispensable to correct completely the absolute concentration value.

The technical limitations of the system, particularly in the case of CH_4_, lead us to the conclusion that the PAS may not be the optimal device to measure methane under unstable conditions, such as in high mountain grasslands where humidity fluctuations occur. Despite the above discussed technical limitations of the system, the combination of the PAS and the chamber technique allowed us to measure GHGs in remote mountain areas in the Pyrenees, where to our best knowledge data on GHG, particularly CH_4_ and N_2_O, are scarce or even lacking. Once acknowledged the strength and weaknesses of the data, we advocate for the usability of such GHG flux measurements.

Overall, this method derives from a corpus of reviews on chamber-based measurements in the field, but is not following the recommendations where data of lower quality are discarded in order to achieve high data accuracy. Rather, we acknowledge that lower quality data enclose useful information that at least can be assessed in a qualitative manner.

## Figures and Tables

**Figure:**
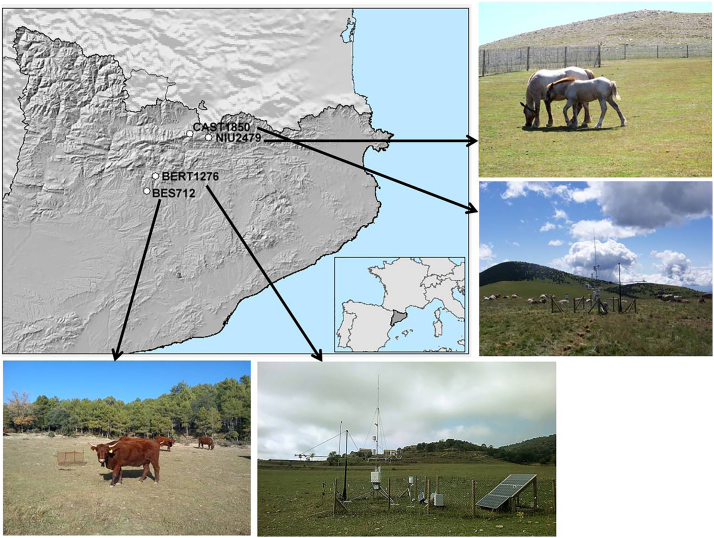


## References

[1] Yamulki S., Jarvis S.C. (1999). Automated chamber technique for gaseous flux measurements: evaluation of a photoacoustic infrared spectrometer- trace gas analyzer. J. Geophys. Res..

[2] Delle Vedove G., Grignani C., Bertora C. (2012). Greenhouse gases emissions from soils. Handb. Stand. Ecosyst. Protoc. – Infrastructures Exp. Ecosyst. Res., Italy.

[3] Oertel C., Matschullat J., Zurba K., Zimmermann F., Erasmi S. (2016). Greenhouse gas emissions from soils—a review. Chem. Der Erde Geochem..

[4] Moody L.B., Li H., Burns R.T., Xin H., Gates R.S., Hoff S.J., Overhults D.G. (2008). A quality assurance project plan for monitoring gaseous and particulate matter emissions from broiler housing. Am. Soc. Agric. Biol. Eng..

[5] Castellano M.J., Schmidt J.P., Kaye J.P., Walker C., Graham C.B., Lin H., Dell C.J. (2010). Hydrological and biogeochemical controls on the timing and magnitude of nitrous oxide flux across an agricultural landscape. Glob. Change Biol..

[6] Iqbal J., Castellano M.J., Parkin T.B. (2013). Evaluation of photoacoustic infrared spectroscopy for simultaneous measurement of N2O and CO2 gas concentrations and fluxes at the soil surface. Glob. Change Biol..

[7] Wang Z.P., Gulledge J., Zheng J.-Q., Liu W., Li L.-H., Han X.-G. (2009). Physical injury stimulates aerobic methane emissions from terrestrial plants. Biogeosciences.

[8] Kutzbach L., Wille C., Pfeiffer E.-M. (2007). The exchange of carbon dioxide between wet arctic tundra and the atmosphere at the Lena River Delta, Northern Siberia. Biogeosci. Discuss..

[9] Parkin T.B., Venterea R.T., Follet R.F. (2010). Chapter 3: chamber-based trace gas flux measurements. Sampl. Protoc..

[10] Pedersen A.R., Petersen S.O., Schelde K. (2010). A comprehensive approach to soil-atmosphere trace-gas flux estimation with static chambers. Eur. J. Soil Sci..

[11] Tirol-Padre A., Rai M., Gathala M., Sharma S., Kumar V., Sharma P.C., Sharma D.K., Wassmann R., Ladha J. (2014). Assessing the performance of the photo-acoustic infrared gas monitor for measuring CO2, N2O, and CH4 fluxes in two major cereal rotations. Glob. Change Biol..

[12] Dengel S., Levy P.E., Grace J., Jones S.K., Skiba U.M. (2011). Methane emissions from sheep pasture, measured with an open-path eddy covariance system. Glob. Change Biol..

[13] Merbold L., Eugster W., Stieger J., Zahniser M., Nelson D., Buchmann N. (2014). Greenhouse gas budget (CO2, CH4 and N2O) of intensively managed grassland following restoration. Glob. Change Biol..

[14] Blankinship J.C., Brown J.R., Dijkstra P., Hungate B.A. (2010). Effects of interactive global changes on methane uptake in an annual grassland. J. Geophys. Res..

[15] Imer D., Merbold L., Eugster W., Buchmann N. (2013). Temporal and spatial variations of soil CO_2_, CH_4_ and N_2_O fluxes at three differently managed grasslands. Biogeosciences.

[16] Nicoloso R.D.S., Bayer C., Denega G.L., De Oliveira P.A.V., Higarashi M.M., Corrêa J.C., Lopes L.D.S. (2013). Gas chromatography and photoacoustic spectroscopy for the assessment of soil greenhouse gases emissions. Ciên. Rural.

[17] Pihlatie M.K., Christiansen J.R., Aaltonen H., Korhonen J.F.J., Nordbo A., Rasilo T., Benanti G., Giebels M., Helmy M., Sheehy J., Jones S., Juszczak R., Klefoth R., Lobo-do-Vale R., Rosa A.P., Schreiber P., Serça D., Vicca S., Wolf B., Pumpanen J. (2013). Comparison of static chambers to measure CH4 emissions from soils. Agric. For. Meteorol..

[18] Ribas A., Llurba R., Gouriveau F., Altimir N., Connolly J., Sebastià M.T. (2015). Plant identity and evenness affect yield and trace gas exchanges in forage mixtures. Plant Soil.

[19] Pirk N., Mastepanov M., Parmentier F.-J.W., Lund M., Crill P., Christensen T.R. (2016). Calculations of automatic chamber flux measurements of methane and carbon dioxide using short time series of concentrations. Biogeosciences.

[20] Luan J., Wu J. (2014). Gross photosynthesis explains the “artificial bias” of methane fluxes by static chamber (opaque versus transparent) at the hummocks in a boreal peatland. Environ. Res. Lett..

[21] Parkin T.B., Venterea R.T. (2010). Chamber-based trace gas flux measurements. USDA-ARS GRACEnet Proj. Protoc. 2010.

[22] De Klein C., Harvey M. (2015). Nitrous Oxide Chamber Methodology Guidelines, Version 1.

[23] Porter P.S., Ward R.C., Bell H.F. (1988). The detection limit. Environ. Sci. Technol..

[24] Langford B., Misztal P.K., Nemitz E., Davison B., Helfter C., Pugh T.A.M., MacKenzie A.R., Lim S.F., Hewitt C.N. (2010). Fluxes and concentrations of volatile organic compounds from a South-East Asian tropical rainforest. Atmos. Chem. Phys..

[25] Helsel D.R. (1990). Less than obvious: statistical treatment of data below the detection limit. Environ. Sci. Technol..

[26] Croghan C.W., Egeghy P.P. (2003). Methods of Dealing with Values Below the Limit of Detection Using SAS, RTP, NC.

[27] Lawrence A.F., Grant R.H., Boehm M.T., Heber A.J., Wolf J.M., Cortus S.D., Bogan B.W., Ramirez-Dorronsoro J.C., Diehl C.A. (2009). Measurements of air quality around various open area sources in US. Int. J. Civ. Environ. Eng..

[28] Ni J.-Q., Heber A.J., Darr M.J., Lim T.T., Diehl C.A. (2009). Air quality monitoring and data acquisition for livestock and poultry environment studies. Trans. ASABE.

[29] Leytem A.B., Dungan R.S., Bjorneberg D.L., Koehn A.C. (2010). Emissions of ammonia, methane, carbon dioxide, and nitrous oxide from dairy cattle housing and manure management systems. J. Environ. Qual..

[30] Rosenstock T.S., Diaz-Pines E., Zuazo P., Jordan G., Predotova M., Mutuo P., Abwanda S., Thiong’O M., Buerkert A., Rufino M.C., Kiese R., Neufeldt H., Butterbach-Bahl K. (2013). Accuracy and precision of photoacoustic spectroscopy not guaranteed. Glob. Change Biol..

[31] Flechard C.R., Neftel A., Jocher M., Ammann C., Fuhrer J. (2005). Bi-directional soil/atmosphere N2O exchange over two mown grassland systems with contrasting management practices. Glob. Change Biol..

[32] Neftel A., Flechard C., Ammann C., Conen F., Emmenegger L., Zeyer K. (2007). Experimental assessment of N2O background fluxes in grassland systems. Tellus Ser. B: Chem. Phys. Meteorol..

[33] Predotova M., Gebauer J., Diogo R.V.C., Schlecht E., Buerkert A. (2010). Emissions of ammonia, nitrous oxide and carbon dioxide from urban gardens in Niamey, Niger. Field Crops Res..

