# Supplementary material for: Maximizing the information obtained from chamber-based greenhouse gas exchange measurements in remote areas
Source: MethodsX. 2018 Aug 22;5:973–83. doi: 10.1016/j.mex.2018.07.021 (PMC6120723; doi:10.1016/j.mex.2018.07.021)
Supplement: Supplementary file 1 [file mmc1.docx]

# Additional information:

Mountain ecosystems are particularly sensitive to climate change because the seasonality of runoff in snow-melt dominated regions is directly, and at a faster rate, affected by warmer climate compared to other areas [1]. In fact, these ecosystems are witnessing an increase in the atmospheric carbon dioxide (CO_2_) concentration and other greenhouse gases, and a parallel increase in global mean surface temperatures. In the Pyrenees, these effects of warming have been shown to be particularly pronounced [2,3]. Assessing greenhouse gases (GHG) exchange in mountain grasslands, particularly in the Pyrenees, is crucial since these ecosystems can act as both sinks and sources of carbon dioxide [4] depending on resource availability and land use practices.

In this context, we measured vegetation and soil fluxes of CO_2_, CH_4_, and N_2_O from four grassland locations along an altitudinal gradient in the Eastern Pyrenees (Fig. S1). The four locations are distributed along an altitudinal and climatic gradient in the South-Eastern Pyrenees and represent typical extensively grazed semi-natural mountain grasslands. The four locations are: Besora (712 m a.s.l.; BES712; 42º1'6.40"N, 1º36'19.54"E), La Bertolina (1276 m a.s.l.; BERT1276; 42º5'50.66''N, 1º39'44.68''E), Castellar de n’Hug (1850 m a.s.l.; CAST1850; 42º18'12.19''N, 2º1'53.60''E), and Niu de l’Àliga (2479 m a.s.l.; NIU2479; 42º19'12.24''N, 1º54'0.49''E).

The common sampling strategy was based on periodic one-day visits to the sites to collect chamber-based flux data and biomass. The measurements took place during daylight (between 8 am and 4 pm approximately; solar time). The sites were visited at four-to-six-week intervals (depending on the length of the growing season at each site) in order to represent the different stages of development.

"Figure S1. Locations of the four study sites: Besora (BES712), La Bertolina (BERT1276), Castellar de n’Hug (CAST1850), and Niu de l’Àliga (NIU2479)."

Sampling points were spread over a grid in the grassland; the diameter of the circular samples represented by metallic collars was 25 cm. At BERT1276 and CAST1850, the chamber sampling points were placed inside the footprint of the environmental-data measuring equipment, a target area of about 50-70 m radius. At BES712 and NIU2479, the sampling points were spread over a grid on the grassland, covering a similar area. The points were chosen anew for every sampling date because of destructive biomass sampling, and chambers were placed over the collars, to obtain flux measurements.

The campaigns differed in 2012 and 2013 in the following ways: In 2012 the points were arbitrarily distributed on the grassland (N=9 per site), and only measures with intact vegetation under light and dark conditions were performed. In 2013, the points were chosen to represent the dominant plant functional types (PFT), -grasses, sedges, legumes, and non-legume forbs-, with three replicates per PFT (thus N= 9 or 12 per site). Therefore, we had a total of nine to twelve sampling points per measurement campaign and site, depending on whether the site was dominated by three or four PFT. Also, during 2013, additionally to the flux measures with intact vegetation in light and dark, soil dark measurements were taken after the vegetation had been harvested.

# References:

[1] IPCC, CLIMATE CHANGE 2013 - The Physical Science Basis. Contribution of Working Group I to the Fifth Assessment Report of the Intergovernmental Panel on Climate Change, Cambridge University Press, Cambridge, United Kingdom and New York, NY, USA, 2013. doi:10.1017/CBO9781107415324.

[2] J. López-Moreno, Climate change prediction over complex areas: spatial variability of uncertainties and predictions over the Pyrenees from a set of regional climate models, Int. J. Climatol. 1550 (2008) 1535–1550. doi:10.1002/joc.

[3] J.I. López-Moreno, M. Beniston, Daily precipitation intensity projected for the 21st century: seasonal changes over the Pyrenees, Theor. Appl. Climatol. 95 (2009) 375–384. doi:10.1007/s00704-008-0015-7.

[4] M. Galvagno, G. Wohlfahrt, E. Cremonese, M. Rossini, R. Colombo, G. Filippa, T. Julitta, G. Manca, C. Siniscalco, U. Morra di Cella, M. Migliavacca, Phenology and carbon dioxide source / sink strength of a subalpine grassland in response to an exceptionally short snow season, Environ. Res. Lett. 8 (2013). doi:doi:10.1088/1748-9326/8/2/025008.
